# Onset and Seasonal Kinetics of Xylogenesis in *Pinus sylvestris* L. on the Southern Fringes of Its Distribution Depend on Early Spring Air and Soil Temperature

**DOI:** 10.3390/plants15131933

**Published:** 2026-06-23

**Authors:** Liliana V. Belokopytova, Natalia V. Karmanovskaya, Dina F. Zhirnova, David M. Meko, Yulia A. Kholdaenko, Elena A. Babushkina, Eugene A. Vaganov

**Affiliations:** 1 Institute of Ecology and Geography, Siberian Federal University, Krasnoyarsk 660041, Russia; dina-zhirnova@mail.ru (D.F.Z.); kropacheva_yulechka@mail.ru (Y.A.K.); babushkina70@mail.ru (E.A.B.); eavaganov@hotmail.com (E.A.V.); 2 Khakass Technical Institute, Siberian Federal University, Abakan 655017, Russia; 3 Ecological Educational Center “Noosphere”, Fedorovsky Polar State University, Norilsk 663310, Russia; karmanovskayanv@norvuz.ru; 4 Laboratory of Tree-Ring Research, University of Arizona, Tucson, AZ 85721-0045, USA; dmeko@arizona.edu; 5 Department of Dendroecology, V.N. Sukachev Institute of Forest, Siberian Branch of the Russian Academy of Science, Krasnoyarsk 660036, Russia

**Keywords:** Scots pine, phenology of wood formation, cambial activity, xylem differentiation, soil thermal regime, threshold temperature, snowmelt

## Abstract

Climatic variation is inherently linked with tree phenology; however, phenological triggers depend on species and habitat. We analyzed key climatic factors for the onset of secondary growth for Scots pine (*Pinus sylvestris* L.) at the southern limit of its distribution in Siberia. From direct observations of developing tree rings, seasonal curves of the number of cells in the cambial zone, in the cell-expansion zone, and the total number of xylem tracheids were developed over seven years with a wide variety in the phenological dates. We found that later and shorter intervals of these stages of xylogenesis were compensated by higher maximums of kinetics curves, probably due to higher temperatures and daylengths during the respective phenophases. Air temperature and soil temperature at a depth of 20 cm converged to values (mean ± SE) 6.6 ± 0.9 °C (air) and 3.7 ± 0.4 °C (soil) for a 15-day interval prior to cambial activity onset. Date of Tsoil ≥ 3.5 °C was most closely related to cambial activity onset (*r* = 0.99) and preceded it by 9.6 ± 1.1 days. Cumulative temperature sums were less reliable. Apparently, both air and soil temperature thresholds have to be reached for cambial division to start in this species-habitat combination. Late abundant snowfall can yield divergence between air and soil temperatures and delay the onset of xylogenesis.

## 1. Introduction

Climate fluctuations and their long-term dynamics influence the phenology of many tree growth processes [1,2,3,4]. The timing of phenological events is a major determinant of plant productivity and species distribution [5,6]. Temperature is noted to be the primary trigger for many seasonal plant growth processes [7,8,9,10]. In the first half of the growing season, phenological shifts can be associated with an earlier or later onset of spring, snowmelt, and warming of the soil and air [11,12,13].

With regard to secondary growth of woody plants, some authors emphasize the critical importance of air temperature in regulating the xylogenesis process and results, while others highlight the input of soil temperature [14,15,16,17,18,19]. Despite the existence of studies outlining the air temperature limits for the onset of xylogenesis in conifers on a global scale [20,21], it is obvious that particular thresholds can be species-specific and modified by natural and climatic conditions [13,22]. It is also known that long-term heat supply, expressed as the sum of positive or active temperatures (above zero or other threshold), can be important for initiating biological processes in plants [23,24].

An earlier onset of xylogenesis can lead to a longer duration and/or higher rate of xylem cell production [25,26]. Understanding this dependence is important because, as noted previously, the rate of cell production and the duration of xylogenesis processes can make different contributions to the final variability in growth [27,28,29].

Key factors driving cambium reactivation in spring are better investigated and understood in cold alpine and boreal areas, given the limitation of tree growth by temperature throughout the vegetation season (e.g., [14,15,20]). There are also some studies of habitats with gradients of growth limitations—either considering elevation transect or performing more global meta-analysis for various regions and biomes [13,19,21,26]. But generally, southern and lower fringes of many boreal tree species’ distributions, where over the main part of the vegetation season, temperature is high enough to act negatively through limiting water supply and even overheating, are less studied at the scale of xylogenesis processes and their regulation. The question is whether heat supply is still a factor kick-starting tree growth in such regions and in what form. And this question is made more relevant since in the warming world, the limitation of tree growth by droughts and excessive heat strengthens and propagates to earlier unaffected regions [7,8].

Since 2013, the Laboratory of Dendroecology and Environmental Monitoring (Khakass Technical Institute of the Siberian Federal University) has been monitoring the seasonal formation of tree rings in southern Siberia [30,31]. Accumulated direct observations of tree ring growth over several years and daily climate series allowed us to ask a fundamental question: what specific temperature threshold is crucial for initiating cambial activity and xylem differentiation in Scots pine (*Pinus sylvestris* L.) growing under conditions of moderate moisture deficit?

## 2. Results

### 2.1. General Shapes of Seasonal Kinetics Curves and Estimations of Phenological Dates

**Figure 1:**
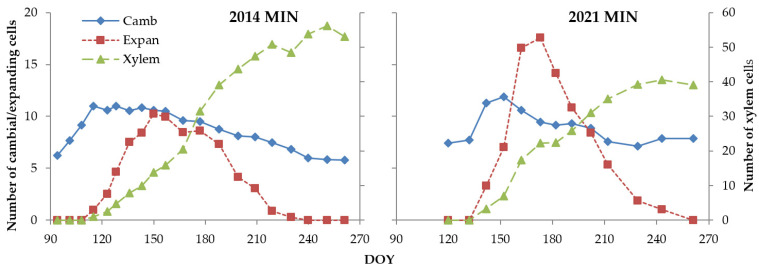
Seasonal kinetics of tree-ring formation on examples of 2014 and 2021, MIN site, from 90th to 270th day of year (DOY): number of cells in the cambial zone (solid line, diamonds), cell expansion zone (dotted line, squares), and xylem cells (dashed line, triangles). Markers indicate the dates of actual observations.

The timing of phenological events was estimated using the annual average curves (the counts of cells on particular stages during the growth season) (Figure 1). In the cambial zone, the kinetic curve shows a relatively stable cell count during the dormant period (varying slightly between the observed radii) and a uni- or bi-modal increase during the active season, with a rapid rise and a slow decline. This curve was used to estimate the onset of cell division and the maximum cambial activity (the observation date with the highest cell count in the cambial zone). The end of cambial activity is difficult to assess from this curve, since its slow decline at the end of the season is lost against the background of variation between radii.

Cells in the expansion zone are characterized by a uni- or bimodal curve with distinct beginning and end points, the dates of which were estimated, and zero values beyond these points. The maximum cell count in this zone (the date of a specific observation) was also recorded.

The beginning of the curve for total xylem cell count and cells in the expansion zone coincide, but then the xylem cell count continues to increase until a conventional plateau (taking into account variations between radii). The point at which the plateau is reached represents an estimated end of cambial activity; this date can be visually refined, if necessary, by the appearance of a distinctive cell size difference between the cambial zone and xylem tracheids.

Obviously, these estimates of phenological dates are imprecise, but their errors should be expected within half the time between actual observations, i.e., less than a week for the first half of the season.

### 2.2. Characteristics of Xylogenesis Phenology in Years with Early and Late Cambium Activity

The full variability range of onset dates over seven considered years was found to be about a month (31–35 days); for maximum and ending dates, it was about three weeks (19–24 days) (Table 1).

Visual analysis of cell number kinetics in individual zones of developing tree rings in different years clearly revealed that in 2013, 2014, and 2017, the increase in cambium cell numbers occurred earlier than in 2018, 2019, 2021, and 2023. For brevity, these year subsets are referred to from here on as the “early” and “late” groups. This difference in the increase of cambial cell numbers between groups is significant and is followed by differences in subsequent zones of tree-ring formation. For example, after standardization of the curves, the cambial cell number is significantly (*p* < 0.05) higher from 4 May to 20 May for the “early” group than for the “late” group. The curves for the cell expansion zone are significantly higher for the “early” group than for the “late” group from 10 May to 4 June. Conversely, from 12 June to 14 July, expanding cells are significantly more numerous in the “late” group than in the “early” group. For the standardized cumulative xylem cell count curves, significantly higher values in the “early” group are observed from 9 May to 2 June.

The onset and maximum of activity in the cambial zone and in the cell expansion zone (i.e., the period of increase in the number of cells in these zones) are consistently different in the “early” and “late” groups. The difference averages 17–20 days, and is significant or almost significant at *p* < 0.05 (Table 1). On average, 19 April and 7 May are the onset of cell division, 28 April and 16 May are the onset of differentiation, 13 May and 30 May are the maximum of cambial cells, and 1 June and 21 June are the maximum of expanding cells in the “early” and “late” groups, respectively.

Cessation of cambial activity has a much less pronounced difference in timing between “early” and “late” groups: 8 days for the cessation of cell production (17 August and 25) and 6 days for the last cell to emerge from the expansion zone (28 August and 3 September). For both processes, the difference is statistically insignificant. Thus, the duration of cell division and expansion processes is shorter in years with late xylogenesis, but only the deceleration phase of these processes after reaching their maximum is shortened.

The delay of the first xylem cell entering the expansion zone (onset of cell differentiation) after the onset of cell division in the cambial zone averages 9.7 ± 1.6 days (mean ± SE).

**Figure 2:**
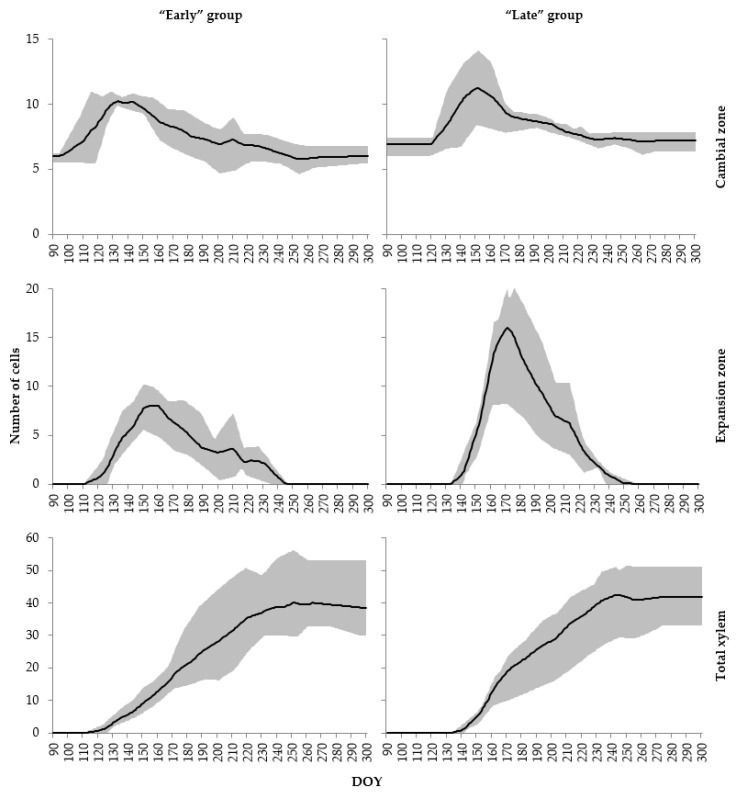
Seasonal kinetics of tree-ring formation, generalized for years with early (2013, 2014, 2017) and late (2018, 2019, 2021, 2023) active xylogenesis: number of cells in the cambial zone, cell expansion zone, and total xylem. The line is the mean value; the shaded area shows the range of variability (min–max).

In the dormant state, the cambial zone consistently consists of 5–7 cells at both the beginning and end of the warm season (Figure 2); at peak cambial activity, it averages 10.9 cells (ranging from 8.5 to 14). After the peak, the cambial cell count decreases slowly and gradually in all years, which is why this curve does not provide a reliable estimate of the cessation of cell production.

**Figure A1:**
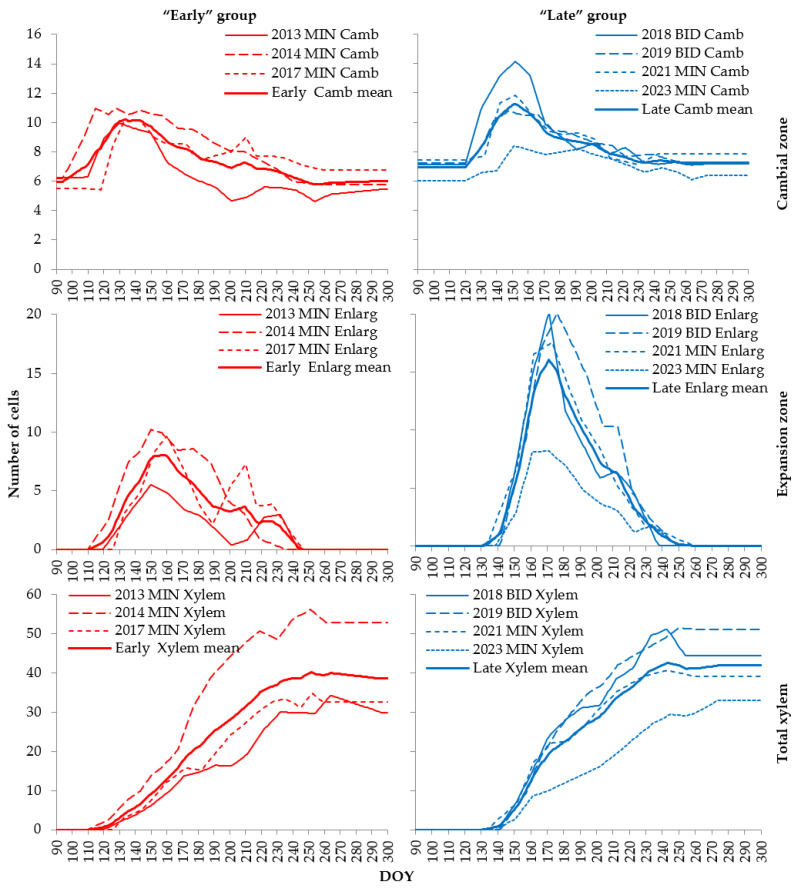
Seasonal kinetics of tree-ring formation for years with early (2013, 2014, 2017) and late (2018, 2019, 2021, 2023) active xylogenesis at sites MIN and BID: number of cells in the cambial zone, cell expansion zone, and total xylem. The thick line is the mean value for a group of years; the thin lines represent kinetic curves for particular years.

For the cell count curve in the expansion zone, a late onset can be compensated for by a more intense differentiation: the peak cell count in years with early xylogenesis is 6–10 cells, while it is 8–20 cells in years with late xylogenesis. Also, in years with early xylogenesis, this curve is characterized by more pronounced asymmetry and even bimodality (Figure A1), while in years with late xylogenesis, the peaks are closer together, usually forming a slightly asymmetric unimodal curve.

The final xylem cell count per radial row in the formed tree ring is quite high and within the same range for the selected years with early and late xylogenesis (42–44 on average for groups, 33–56 in particular years). This means that all these years were relatively favorable for the radial growth of pine trees at the sampling sites. Therefore, the later onset of the cumulative curve is compensated for within a month, and the subsequent differences between the groups are insignificant.

Note that for 2017 and 2018, despite the close dates of cell division onset (a 2-day difference), the subsequent course of the kinetic curves in their first half differs sharply: in 2018, the onset of cell expansion was observed 5 days later than in 2017, the maximum of cells in the cambial zone 9 days later, and the maximum of cells in the expansion zone 11 days later than in 2017. Therefore, these years were separated into different samples based on the aggregate pine growth kinetics.

### 2.3. Key Temperature Factors for Initiating Xylogenesis

No significant patterns were found for precipitation amounts during the period preceding cambium activation. Air temperature (Tair) was generally positively associated with the onset of cambial activity. However, the sharp difference in xylogenesis kinetics between 2017 and 2018, despite similarities in its onset and air temperature (2018 had a warm spring, similar to the years in the “early” group), led us to search for other climatic factors that inhibited the first stages of xylogenesis in 2018.

It turned out that soil temperature (Tsoil) in the spring of 2018 was significantly colder than in the years from the “early” group. For example, on April 19, daily soil temperature at a depth of 20 cm for the “early” group was 3.1–7.3 °C, while in 2018 it was only 1.7 °C, which is closer to the other years in the “late” group (0.6–1.2 °C). After smoothing with the 15-day moving average, soil temperature for this day was 4.7–6.6, 2.9, and 1.3–2.2 °C for the “early” group, 2018, and the rest of the “late” group, respectively. The reason for such slow soil warming in 2018 was discovered to be abnormal snow cover. The last significant snowfalls that year occurred on 31 March and 1 April (24 cm of snow), accompanied by frosts down to –10 °C. Subzero daily air temperature continued until April 6, and the snow did not melt until 9 April. Of all the years analyzed, these are the latest dates for significant snow cover lasting more than three days after falling. Throughout all of March 2018, the snow cover was also the most stable and deep: 8.4 cm on average, and only 3 days with bare soil, while for the other years considered, March snow cover averaged 1.7 cm and was absent for 10–30 days in both groups.

Therefore, in addition to air temperature, all subsequent analyses were also conducted for soil temperature at a depth of 20 cm. Air and soil temperature dynamics as potential key factors were analyzed by aligning their seasonal curves with our estimate of the onset of cell division in the cambial zone for each year, similar to the superimposed epoch method used in dendrochronology and climatology [32], but on an intraseasonal scale with a daily resolution. This allows us to identify patterns common to all years considered.

**Figure 3:**
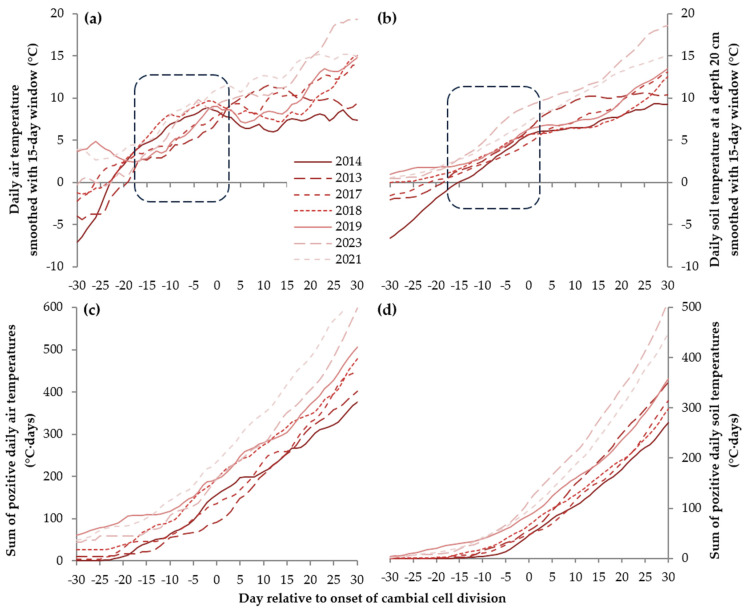
Dynamics of air and soil temperature relative to the onset of cell division in the cambial zone, defined as day 0. (**a**) Daily air temperature. (**b**) Daily soil temperature at a depth of 20 cm. (**c**) Sum of positive air temperatures. (**d**) Sum of positive soil temperatures at a depth of 20 cm. Curves in (**a**,**b**) are smoothed by a 15-day moving average. The shade gradient of the temperature curves is from the earliest (dark) to the latest (light) date of the onset of xylogenesis. Dashed rectangles highlight periods of high similarity among the temperature curves for all the years.

Smoothed curves of daily air and soil temperature dynamics (Figure 3a,b) show that inter-year variability is larger 30 days before the onset of cambial activity and 20–30 days after this phenological date. In contrast, during the 15–20 days before cambium activation, both temperature curves converge among years into tighter “bundles.” During this time interval, there was no pattern in the temperature curve’s dependence on the calendar date of xylogenesis onset, but before and after this interval, the “late” group experienced higher temperatures than the “early” group.

Next, the dynamics of the cumulative sums of positive temperatures accumulated since the beginning of calendar spring (i.e., excluding possible winter thaws) were examined (Figure 3c,d). For these indicators, consistently positive correlations with the calendar dates of the first phases of xylogenesis were observed throughout the entire 61-day interval examined, with the variability of the sum of temperatures steadily increasing over time. However, it should be noted that at and before the onset of xylogenesis, the variability of cumulative soil temperature is less than that of air.

A summary of air and soil temperature for the “early” and “late” groups shows (Table 2) that during the last 16 days before the reference point (the first cell division) the standard error or temperature does not exceed 0.6 °C, while before the beginning of this interval, 24 days before the reference point, the SE reaches 0.7–0.9 °C, and during the 24 days after the onset of xylogenesis, SE gradually increases to 1.0–1.3 °C. Taking into account the centered 15-day temperature smoothing window we used, the value of the curve 8 days before the reference point actually corresponds to the average temperature for 15 days directly preceding the onset of cambial cell division. It should be noted that the average temperature during this interval was ~6.6 °C for the air and ~3.7 °C for the soil, with positive temperature sums of 103 and 30 °C·day, respectively. Meanwhile, at the onset of cell division, plus or minus one week, the average temperature was 8.9 and 6.4 °C for air and soil, respectively.

The most consistent temperature thresholds were observed for the 15-day interval before the onset of xylogenesis. Thus, after rounding these identified threshold values to 0.5 °C (for daily series) or 10 °C (for sums), we investigated calendar dates of stable transition through them for air and soil temperature and their sums. Additionally, we considered dates of soil thaw. A summary of all these dates for “early” and “late” groups of years is presented in Table 1.

The dates of soil thaw are practically the same between the “early” and “late” groups: the difference between the means of 4 days is not statistically significant and is less than the range of variability in each sample. For the dates of stable transition of air temperature through the threshold Tair ≥ 6.5 °C, a difference of 16 days is observed between groups. This difference is not significant (*p* = 0.077), but it is closer to the difference in the phenology of xylogenesis. The accumulated sum of positive temperatures reaches the threshold ΣTair ≥ 100 °C day on average 10 days earlier in the “early” group than in the “late” group; this difference is also insignificant. The dates of stable transition of soil temperature above the threshold of Tsoil ≥ 3.5 °C can differ from the dates of transition of air temperature above Tair ≥ 6.5 °C in individual years, but have the same mean values for both groups. However, for soil temperature, the difference of 16 days between groups is statistically significant. Finally, the sum of positive soil temperatures reaches 30 °C·day 13 days earlier in the “early” group than in the “late” group, and this difference is also significant.

Thus, among the identified thresholds, soil temperature indicators are more closely and consistently associated with xylogenesis onset dates than respective air temperature indicators, and smoothed daily temperature has an advantage over accumulated temperature sum. The timing of soil thaw to a depth of 20 cm is almost unrelated to xylogenesis activation, in contrast to the higher threshold of Tsoil ≥ 3.5 °C. The delay of xylogenesis onset from the climate threshold is also most consistent for Tsoil ≥ 3.5 °C (Table 3); for the other temperature indicators, the delay is much more variable year to year.

The date of the first xylem cell’s transit to differentiation (expansion) has less stable and significant relationships with the timing of temperature transitions and their sums than the date of cell division onset. However, for this phenological event, the most stable relationship and delay is also observed for the climate threshold Tsoil ≥ 3.5 °C. Overall, this particular temperature indicator significantly influences all the phenological dates examined, although this relationship gradually weakens over the course of the season.

As an additional analysis to check if differences between sites (spatial temperature variation, habitat and stand traits, etc.) confounded our findings, we repeated calculations shown in Table 3 for the MIN subsample of five years (Table A1). Despite lower sample depth, we found the same pattern: climatic date when Tsoil reaches 3.5 °C being the most reliable temperature variable regarding connections with phenological dates. Both air and soil current temperature thresholds also proved to provide better results than respective cumulative sums. For example, the delay of cell division onset after soil temperature reaches the threshold 3.5 °C is 9.6 ± 1.5 (5 to 13) days, for cell expansion delay is 18.0 ± 1.5 (14 to 22) days, their SE and full range of variation are the least among all considered temperature variables. Correlations between climatic and phenological dates are the similar or higher within one sampling site: 0.77–0.92 for Tair ≥ 6.5 °C, 0.72–0.89 for ΣTair ≥ 100 °C·day, 0.61–0.94 for soil melting point, 0.90–0.99 for Tsoil ≥ 3.5 °C, and 0.85–0.97 for ΣTsoil ≥ 30 °C·day, and only for current soil temperature thresholds all correlations are significant at *p* < 0.05.

## 3. Discussion

### 3.1. Seasonal Kinetics of Cambial Activity Initiation and Its Methodological Uncertainties

The process of initiation of tree-ring formation consists of several stages, beginning sequentially or, in some cases, in parallel, and is observable to varying degrees using electron or light microscopy [33,34]. On an intracellular scale, reactivation (emergence from dormancy) of the cambial initial cell is observed through changes in its ultrastructure and, later, the appearance of mitotic figures [35,36], with the time difference between these stages being up to a month [37]. The same pattern of reactivation is also typical for the overwintered xylem and phloem mother cells located on either side of the initial and virtually indistinguishable from it. However, reactivation of the mother cells can either precede or lag the reactivation of the initial itself. In the first case, it is even possible for the differentiation of the first xylem cells located at the very border of the cambial zone to begin before the onset of cell division [34,38]. However, more often, a delay of several days to a month is observed for the onset of cell differentiation after the onset of cell division. This behavior was described more than half a century ago for various tree species, for example, by Evert et al. [36,39,40,41,42].

At the scale of observations using light microscopy, where intracellular structures are not visible, the initiation of xylem formation is traced by the presence of newly formed, very thin tangential walls between cells in the cambial zone, and indirect indicators: an increase in the cambial cell number and the presence of differentiating cells near the cambial zone. However, variability in the relationship between the timing of the onset and the dynamics of the intensity for cell division and cell exit from the cambial zone for differentiation leads in some cases to the absence of an observable increase in the number of cambial cells within a season [43], or delay of this increase after the onset of cell division by 2–4 weeks [36,40,41,44]. This probably depends on the ratio of production of various hormones and the expression of genes that regulate cell division and differentiation [45,46].

Thus, direct intracellular and indirect indicators of xylogenesis initiation—cell reactivation, cambial activity (division of initial and mother cells), and the onset of xylem cell differentiation—may have varying temporal relationships. Therefore, even when observing solely with light microscopy, as in this study, it is reasonable to consider both the number of cambial cells and the onset of xylem differentiation (cell growth by expansion) as signs of an active xylogenesis. When interpreting our results, it is important to keep in mind these methodological uncertainties and the existence of preceding stages of cambial cell awakening that can be observed using electron microscopy.

### 3.2. Observed Timing and Intensity of Xylogenesis Kinetics

This study was initially motivated by the observation, from multi-year seasonal monitoring of the kinetics of tree-ring formation, that some years had contrasting early and late onsets and peaks of cambial activity. The average difference between years with early and late onset of xylogenesis, both in cambial cell division and in differentiation, was 18 days. This range of variation in the timing of the onset of seasonal growth is consistent with published seasonal observation data and climate model calculations [14,21,47,48]. The difference in the timing of peak cell numbers in the cambial zone and in the expansion zone also falls within the range 17–20 days. If these differences, as well as the differences between the onset of cambial activity and the transition of the first cell to the expansion zone, are actually linked to the functioning of the cambial initial, this may be one of the kinetic invariants in the seasonal formation of tree rings, which can be widely used in process models of cambial activity [49,50,51,52,53]. Then the increase in the number of cambial cells will be determined by the number and activity of mother cells. Obviously, at least two options for increasing cell production arise: an increase in the number of dividing mother cells in the cambial zone (its widening) or an increase in the rate of divisions, i.e., the rate of cell cycle progression [54]. Our data obtained for different years support the first option. Thus, the higher maximum number of cambial cells and, as a consequence, the number of expanding cells in years with a late xylogenesis compared with early xylogenesis shows the potential for compensatory growth, which is ultimately aimed at reducing the variation in the total production of tracheids during the season [27,35].

The first cells in the expansion zone appear, on average, 9–10 days after the onset of cell growth in the cambium in both “early” and “late” groups, with the overall delay varying from 3 to 16 days. This is shorter than previously published estimates of the initial cell cycle [35,55]. Thus, it can be assumed that the “pushing” of the peripheral cell from the cambial zone into the xylem differentiation zone (growth by expansion) in Scots pine under these conditions occurs during the first cell cycle after cambium initiation. Regarding the timing of maximums in the early stages of xylogenesis, it should be noted that the maximum cell count in the expansion zone is reached around the summer solstice, even in years with late xylogenesis. This means that the period of most active cell division in the cambial zone and the initiation of their differentiation occurs entirely during the part of the season with lengthening daylight hours. However, these phenological events (the activation and increasing intensity of cell division and expansion) consistently occur under greater insolation in years with a late onset of xylogenesis than in years with an early onset. The slowdown in cambial activity and radial growth overall after the solstice is consistent with other observations for boreal forests [56].

### 3.3. External Regulation of Xylogenesis Phenology

The moderate moisture deficit and the timing of droughts in the study region (mainly in June–July) mean that, unlike drier semi-desert forest stands [57,58,59] or even forest-steppes of south Transbaikalia, which tend to experience spring droughts [60], the soil moisture during the xylogenesis activation period for the stands studied here is apparently sufficient for xylogenesis onset to be regulated by temperature and photoperiod, as in most temperate and boreal forests [61,62,63,64]. However, as our results have shown, precipitation at the end of the climatic winter (March snowfalls) also contributes through its impact on soil temperature.

The threshold air temperature or the accumulation of a certain sum of active temperatures (positive or above some higher threshold) for the activation of xylogenesis, including for conifers, has been studied in recent decades by more than one research team. For example, the importance of temperature for the onset of cell production has been noted by several researchers both under natural conditions and in heating or cooling experiments [65,66,67]. Threshold values of mean daily air temperature for the onset of cell division in the xylem of conifers, varying in the range of 5.6–9 °C, were identified in the works of Rossi et al. [20,22,68,69]. The sums of average daily temperatures exceeding 5 °C and their role in the onset of cell production for Scots pine were considered with varying success in the work of Seo et al. [70], and for European beech in the study by Prislan et al. [71]. In both cases, the relationship was positive, but weak or ambiguous (e.g., varying temperature sums depending on the site), suggesting interaction of this factor with others. On the other hand, growing degree-days and photoperiod were reported to determine the growth onset of Olga Bay larch [24]. Another study showed the species-specific nature of the temperature sum impact on growth onset in the same mixed forest stand [23].

The impact of snow cover and soil temperature dynamics on xylogenesis has been studied primarily at the northern forest line, primarily within the permafrost zone. However, even in cold conditions, observations are far from unambiguous. For example, the influence of snowmelt timing on growth and tree-ring structure, as well as indirectly on the phenology of xylogenesis initiation, was observed for several larch species in the cold conditions of northern Siberia [14,15]. It was also shown that along a longitude gradient, early snowmelt is spatially linked to xylogenesis activation at lower air temperature. For black spruce in boreal forests of Canada, the date of complete snowmelt under natural and artificially heated conditions was found to be positively associated with the timing of an increasing number of cambium cells, but not with subsequent phenological events [16,17]. In montane forests of Swiss mountain pine with a short warm season, the timing of snowmelt and soil warming influenced both the onset of cell expansion and radial growth [19].

The deeper underground, the smoother the soil temperature curve (decoupled from short-term air temperature fluctuation) and the more it is lagged in comparison with air temperatures (see Figure 2 in [18] for the average seasonal curves of air and soil temperatures in the study area). Therefore, the threshold of current soil temperature ≥ 3.5 °C at a depth of 20 cm can be approximately linked to the actual complete unfreezing of all root-inhabited soil layers. This leads to the melting of soil water and a subsequent decrease in its viscosity in the warmer upper layers; resumption of water and nutrient uptake by fine roots happens at this stage, as well as the onset of root growth [72]. Nutrient uptake at this time is also indirectly promoted by soil microorganisms performing nutrient decomposition, as is shown in the same monograph. On the contrary, warm air and cold or frozen soil limit usable water to inner storage, which slows down photosynthesis [73]. Physiological reactions of conifers to cold soil also include decreases in xylem pressure potential and stomatal conductance as mechanisms of suppressing photosynthesis, delayed onsets of shoot and radial growth [74,75,76,77]. However, compensation of growth delay by its faster rate after sufficient warming was also observed, in particular, for Scots pine seedlings [76,77].

In the semiarid stands of Siberian larch in our study region, it was found that not only air temperature but also the temperature of the root-inhabited zone of soil (depths of 20–40 cm) negatively impacts growth in May [18], indicating a shift in limitation from heat supply to moisture deficit shortly after the onset of xylogenesis. However, our results suggest that early spring soil temperature positively influences the phenology of cambial zone reactivation.

Both soil and air temperatures exhibit similar dynamics for all the years examined during the 15-day period preceding the observed onset of cell division. The air temperature during this period (6.6 °C) is within the range of threshold temperatures previously identified for the onset of xylogenesis in conifers. The corresponding soil temperature threshold at a depth of 20 cm is also significantly above freezing (3.7 °C), but within the range of significant impact on Scots pine growth. Therefore, we can assume that this critical period for the onset of xylogenesis likely coincides with ultrastructural and chemical changes in cambial cells and is regulated by both the onset of active photosynthesis in warmed air and water uptake by roots from thawed soil. These processes are clearly interconnected, as are the temperatures of air and soil. Therefore, cambial activity likely initiates and accelerates upon reaching both thresholds, as demonstrated by the examples of 2017 and 2018.

An interesting finding from seasonal observations is that crossing the temperature threshold for initiating cambial activity is more significant than the accumulated sum of temperatures (total heat supply). This indirectly points to possible mechanisms regulating growth processes in trees, as well as to a direction for searching for their intracellular regulators [78,79]. Unfortunately, there are still many unknowns about the actual mechanisms by which even temperature influences the initiation of growth processes in trees [52,80]. And how the temperature signal reaches the forming annual ring and modifies (controls) cell production and differentiation remains a mystery.

The mechanism by which the late onset of xylogenesis is compensated for by more intense cell production, resulting in the lack of influence of xylogenesis initiation timing on radial growth, becomes apparent when examining temperature centered on the onset of cambial activity. Figure 3 clearly shows that the later the onset of tree ring formation, the higher the temperature (both current and accumulated) during its most active phase afterward (days 0–30). Combined with later calendar dates in spring, meaning longer daylight hours, this logically leads to a corresponding increase in the availability of photosynthetic products and the intensity of growth processes. However, in the climatic conditions of the study area, the positive effect of temperature can be limited by reversal of its role later within the growth season, when moisture deficit becomes the main growth-limiting factor; thus, photoperiod may be the key factor for the compensatory effect.

A decrease in photoperiod subsequently likely also regulates the reduction in differences in the completion of growth processes, but the reduction in growth rate to zero from the higher maximum characteristic in years with a late onset of xylogenesis still appears to require more time than with an early onset.

## 4. Materials and Methods

The study was conducted in southern Siberia, in a large intermontane basin along the upper reaches of the Yenisei River, where, due to the semiarid continental climate [81], vegetation consists primarily of steppes and agricultural lands. Slightly more humid conditions, suitable for forest growth, are found in the foothills along the valley margins and on sandy soils on the right bank of the Yenisei and its tributaries.

Daily time series of average air temperature, precipitation, soil temperature at a depth of 20 cm, and snow depth for the observation years for the Minusinsk station (53°41′ N 91°40′ E, 250 m a.s.l.) were taken from the database of All-Russian Research Institute of Hydrometeorological Information—World Data Center (http://meteo.ru/data/, accessed on 7 April 2026; [82,83,84]). As our previous research in the study region has shown, the climate dynamics across the study area are synchronous, and radial growth of pine at both sites shows significant responses to climate variables measured at the Minusinsk weather station [85,86]. The choice of a depth of 20 cm for soil temperature is due to the fact that Scots pine has a shallow root system, and 20 cm is a good estimate of the middle of the root-inhabited zone. Depending on habitat and climate, 50% or more of the Scots pine root mass, especially absorptive fine roots, are located at depths of less than 40–50 cm [87,88,89,90], including observations in the study region [90].

In this study, we used temperature series smoothed from daily series by a centered moving average (15-day window and 1-day step were selected empirically), as well as cumulative sums of positive temperatures, starting from March 1. The date of a stable temperature transition through a certain temperature threshold was defined as the first day the threshold was exceeded in the smoothed series, and for which the sum of deviations from the threshold is positive for all subsequent days (this adjustment was made to deal with the case of recurrent cold spells).

Scots pine trees were sampled from two moisture-deficient habitats: (1) a belt forest within the steppe zone, near the city of Minusinsk (MIN, 53°39–40′ N, 91°36–42′ E, 260–320 m a.s.l.); and (2) at the lower forest limit near the village of Vershino-Bidja (BID, 54°00′ N, 90°59′ E, 600–650 m a.s.l.) in the foothills of the Kuznetsk Alatau spurs. The forest stand in habitat MIN is closed, represented by uneven-aged Scots pine on well-drained sandy soils within a flat landscape, with some birch trees growing close to the forest edges. At the BID habitat, the forest stand is sparse, pine-larch with birch, on rocky loamy soils. Sampling at both sites was carried out on sunlit, gentle slopes facing south and east. To observe seasonal growth, from 7 to 15 mature trees without close neighbors or mechanical damage were selected every season (different trees each year).

**Figure A2:**
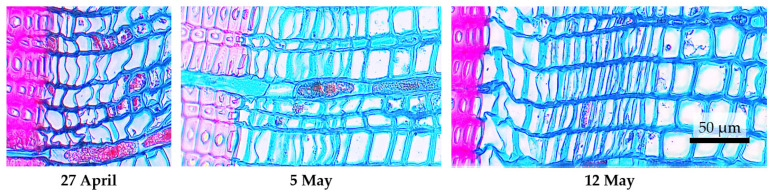
Microphotographs of the developing ring in the beginning stages of xylogenesis on example of MIN sampling site in2017 (the same tree): on 27 April, number of cambial cells (small ones between lignified previous ring and larger phloem cells) is minimal, 5 cells; on 5 May, increase in cambial zone to 9 cells is already observed, in some radial files 1 cell closest to the previous ring begins to expand above cambial cell size; on 12 May, there are 4 differentiating cells in the expansion zone (larger ones) and 11–12 cambial cells (smaller ones) visible between previous ring and phloem. Cell numbers are not necessarily derived from the radial files shown in the figure. The scale bar is the same for all three photographs. Samples were double-stained with astra blue (stains all wood matter) and safranin (red, stains lignin); as a result, magenta color represents lignified cells of previous ring.

In this study, xylogenesis observations for 2013, 2014, 2017, 2021, and 2023 at MIN, and 2018–2019 at BID were used. From late April or early May to mid-September–early October, small wood samples 4.5 mm in diameter containing bark, cambium, a forming annual ring and 2–3 preceding ones were periodically (every 7–20 days depending on the year and season) collected from model trees using a punch tool, with successive collections gradually moving along the trunk in a diagonal direction at a height of 1.2–1.4 m [91,92]. The samples were stored in a mixture of ethyl alcohol, glycerol, and water (1:1:1). Using a Microm HM 340E rotary microtome (Thermo Fisher Scientific, Waltham, MA, USA), 15-μm-thick transverse sections were obtained. The sections were double-stained with astra blue and safranin to highlight lignified tissues [93]. On photographs of sections with a magnification of 400× obtained using a BX43 biological microscope (Olympus, Tokyo, Japan) and a ProgRes Gryphax Subra digital camera (Jenoptik, Jena, Germany), the number of cells in individual zones was counted (Figure A2) and averaged for each date in three radial rows of cells: the cambial zone (the smallest cells between xylem and phloem), the cell expansion zone (larger than cambial cells, but with thin, non-lignified walls) and the total number of xylem cells of the current year (between cambial zone and the border of the previous ring). Counts were made for individual trees and then averaged for each observation date to obtain site-scale kinetics curves.

We did not discern separate cell numbers for further tracheid differentiation (secondary wall thickening and lignification, mature cells) in this study since we focused on the first stages, as most impacted by spring conditions. The total number of xylem cells in all stages of differentiation was used to make more precise estimations for the end of cambial activity.

Trees were not sampled on exactly the same dates in each year. To aggregate data over several years, we first assumed that the cell count in each year was constant before the first observation and after the last observation. Between observations, kinetic curves were estimated daily by interpolation with a piecewise linear function. For the first and last “non-zero” segments of the curve for the cell expansion zone, the slope of the adjacent segment was used. After interpolation, the seasonal kinetics for a group of years was estimated using the arithmetic mean, minimum, and maximum values for each day of year (DOY) from 90 to 300.

To analyze the significance of differences in xylogenesis between groups of years, we standardized the kinetic curves (divided by the minimum value for the cambial zone cell count, by the mean value over DOY 90–300 for the expansion zone, and by the maximum value for xylem cell count) and then tested the differences of means for each DOY during this period using an independent *t*-test.

## 5. Conclusions

Long-term observations of seasonal growth and tree ring formation open up new opportunities for studying both the internal mechanisms of xylem formation and the influence of external conditions. Comparison of climate data for years with early and late onset of xylogenesis in Scots pine in semiarid southern Siberia revealed that the first stage of xylogenesis, reactivation of cambial cells, begins when the average air temperature reaches ~6.5 °C, and the soil temperature reaches ~3.5 °C at a depth of 20 cm. An actual increase in cambial cell number starts on average 8 days after reaching these temperature thresholds. The duration of the increase in cambial cell number after this point is relatively stable, but temperature and daylength regulate its intensity, compensating for the delay.

Future directions of research may include finding underlying physiological mechanisms of temperature impact on cambium reactivation and further processes, including reasons why the current temperature averaged for a 15-day interval seems to be more important for trees than the cumulative heat sum. Also, we hope that further lengthening of the observation series will provide further insights into the Scots pine behavior under a warming climate, considering trends towards shorter winters and longer but less stable spring/autumn seasons. At the moment, this species seems to be well adapted to the current wide range of variability in various temperature thresholds in the study area.

## Figures and Tables

**Table 1:** Estimates of the phenology of pine xylogenesis and the timing of temperature transitions in years with early and late tree-ring formation.

Phenological or Climatic Phenomenon	Calendar Date, Day of the Year (DOY)									*p* *
	Years with Early Active Xylogenesis				Years with Late Active Xylogenesis					
	2013	2014	2017	Mean	2018	2019	2021	2023	Mean	
Xylogenesis of Scots pine										
Onset of cell division in the cambial zone	112	97	120	**110**	122	126	132	130	**128**	0.053
First xylem cell enters expansion zone	120	111	127	**119**	132	142	135	140	**137**	0.020
Maximum cell number in the cambial zone	130	128	143	**134**	152	148	152	151	**151**	0.031
Maximum cell number in the expansion zone	150	150	160	**153**	171	176	173	171	**173**	0.009
Cessation of cambial activity	232	224	235	**230**	233	241	243	234	**238**	0.073
Last xylem cell exits from the expansion zone	243	233	246	**241**	237	247	257	248	**247**	0.150
Temperatures of the environment										
Thawing ** of soil, Tsoil > 0 °C	92	80	89	**87**	89	90	98	89	**91**	0.186
Stable transition *** of air temperature, Tair ≥ 6.5 °C	112	87	105	**101**	108	119	121	121	**117**	0.077
The sum of positive air temperatures, ΣTair ≥ 100 °C·day	113	93	106	**104**	113	105	117	120	**114**	0.118
Stable transition *** of soil temperature, Tsoil ≥ 3.5 °C	105	92	107	**101**	113	116	121	118	**117**	0.033
The sum of positive soil temperatures, ΣTsoil ≥ 30 °C·day	107	95	106	**103**	115	110	120	119	**116**	0.025

* Significance level *p* of the difference between mean values for “early” and “late” groups of years was calculated using an independent *t*-test. ** The first day of Tsoil > 0 °C is defined as the day of soil thaw. *** The dates of stable transition were calculated from the daily temperature series smoothed by a centered 15-day moving average; for recurrent cold spells, the transition was considered stable if the cumulative sum of temperature deviations from the threshold after the transition date is always positive. Mean values for years with early and late active xylogenesis are in bold font.

**Table 2:** Temperature before and after the onset of xylogenesis (mean ± SE).

Day Relative to the Calendar Date of the Onset of Cell Division in the Cambial Zone	Air Temperature Tair, °C		Soil Temperature at a Depth of 20 cm Tsoil, °C	
	Smoothed * W = 15 Days	Cumulative Sum ΣT > 0 °C (°C·Day)	Smoothed * W = 15 Days	Cumulative Sum ΣT > 0 °C (°C·Day)
−24	0.45 ± 0.94	38 ±13	−0.33 ± 0.73	4 ± 2
−16	3.66 ± 0.34	63 ± 12	1.42 ± 0.38	11 ± 4
−8	6.64 ± 0.59	103 ± 14	3.73 ± 0.44	30 ± 7
0	8.88 ± 0.46	172 ± 18	6.42 ± 0.54	73 ± 10
+8	9.12 ± 0.78	244 ± 20	8.01 ± 0.67	135 ± 14
+16	10.20 ± 0.78	320 ± 23	9.30 ± 0.80	203 ± 19
+24	12.38 ± 1.28	410 ± 30	11.70 ± 1.03	286 ± 26

* Smoothed temperature series are centered; e.g., the smoothed temperature on the day of the onset of cell division (day 0) is the daily temperature averaged over days from −7 to +7 inclusive.

**Table 3:** Relationships between dates of phenological and climatic events.

Phenological Event in Pine Xylogenesis		Climatic Event				
		Air Temperature, Tair		Soil Temperature at a Depth of 20 cm, Tsoil		
		Tair ≥ 6.5 °C	ΣTair ≥ 100 °C·Day	Tsoil > 0 °C	Tsoil ≥ 3.5 °C	ΣTsoil ≥ 30 °C·Day
Delay of phenological date after climatic date (days)						
Onset of cell division	mean ± SE	9.4 ± 1.9	10.3 ± 2.8	30.3 ± 3.3	9.6 ± 1.1	9.6 ± 1.9
	min…max	0…15	−1…21	17…41	5…13	2…16
Onset of cell expansion	mean ± SE	19.1 ± 2.3	20.0 ± 3.3	40.0 ± 3.5	19.3 ± 1.5	19.3 ± 2.4
	min…max	8…24	7…37	28…52	14…26	13…32
Correlation coefficients between phenological and climatic dates *						
Onset of cell division		**0.91**	**0.80**	**0.77**	**0.99**	**0.93**
Onset of cell expansion		**0.87**	0.63	0.56	**0.93**	**0.82**
Maximum number of cells in the cambial zone		0.70	0.64	0.55	**0.90**	**0.87**
Maximum number of cells in the expansion zone		0.73	0.51	0.50	**0.89**	**0.79**
Cessation of cambial activity		**0.84**	0.55	**0.85**	**0.87**	0.72
The last cell leaves the expansion zone		**0.82**	0.64	**0.86**	**0.79**	0.71

* Correlation values significant at *p* < 0.05 are bold.

## Data Availability

Raw quantitative data are included in the Supplementary Materials. Processed data and microphotographs will be available on request from the authors.

## Supplementary Materials

The following supporting information can be downloaded at: https://www.mdpi.com/article/10.3390/plants15131933/s1, an archive of raw data on climatic variables and seasonal kinetics of xylogenesis (cell counts for cambial zone, expansion zone, and total xylem on days of actual observations) used in this study.

## Abbreviations

The following abbreviations are used in this manuscript:

SE	standard error
Tair	air temperature
Tsoil	soil temperature at a depth of 20 cm
DOY	day of year

## Appendix A

**Table A1:** Relationships between dates of phenological and climatic events within the MIN sampling site (for five-year subsample).

Phenological Event in Pine Xylogenesis		Climatic Event				
		Air Temperature, Tair		Soil Temperature at a Depth of 20 cm, Tsoil		
		Tair ≥ 6.5 °C	ΣTair ≥ 100 °C·Day	Tsoil > 0 °C	Tsoil ≥ 3.5 °C	ΣTsoil ≥ 30 °C·Day
Delay of phenological date after climatic date (days)						
Onset of cell division	mean ± SE	9.0 ± 2.5	8.4 ± 3.0	28.6 ± 4.5	9.6 ± 1.5	8.8 ± 2.3
	min…max	0…15	−1…15	17…41	5…13	2…14
Onset of cell expansion	mean ± SE	17.4 ± 2.9	16.8 ± 2.5	37.0 ± 4.0	18.0 ± 1.5	17.2 ± 1.6
	min…max	8…24	7…21	28…51	14…22	13…21
Correlation coefficients between phenological and climatic dates *						
Onset of cell division		**0.92**	**0.89**	0.80	**0.99**	**0.9** **7**
Onset of cell expansion		**0.8** **9**	0.88	0.65	**0.9** **6**	**0.** **95**
Maximum number of cells in the cambial zone		0.78	0.73	0.62	**0.9** **2**	**0.8** **9**
Maximum number of cells in the expansion zone		0.77	0.72	0.61	**0.** **92**	**0.** **90**
Cessation of cambial activity		0.83	0.76	**0.** **94**	**0.** **90**	0.85
The last cell leaves the expansion zone		**0.8** **9**	0.83	**0.** **92**	**0.** **95**	**0.** **92**

* Correlation values significant at *p* < 0.05 are bold.
